# Effects of hyaluronic acid on skin at the cellular level: a systematic review

**DOI:** 10.1590/1806-9282.20250208

**Published:** 2025-09-19

**Authors:** Mehmet Uğur Karabat, Mehmet Cudi Tuncer

**Affiliations:** 1 Dicle University, Faculty of Medical, Department of Histology and Embryology – Diyarbakır, Turkey.; 2 Dicle University, Faculty of Medicine, Department of Anatomy – Diyarbakır, Turkey.

## INTRODUCTION

Hyaluronic acid (HA) is a naturally occurring glycosaminoglycan found abundantly in various human tissues, including the skin, eyes, and joints^1-4^. As a vital component of the extracellular matrix (ECM), HA contributes significantly to maintaining tissue hydration, structural organization, and intercellular communication^5,6^. Its unique physicochemical properties, particularly its remarkable capacity to retain water, have drawn increasing interest within the fields of dermatology and esthetic medicine. HA plays a central role in skin homeostasis and is actively involved in essential biological processes such as wound healing, tissue regeneration, and modulation of inflammatory responses. Accordingly, its application in dermatological practice, including both topical formulations and injectable dermal fillers, has significantly advanced therapeutic and cosmetic approaches to skin care^7-9^.

The skin, as the largest organ of the human body, relies on HA for its biomechanical strength and physiological functionality. Present throughout the epidermis, dermis, and hypodermis, HA exerts diverse effects at the cellular level depending on its molecular weight (MW). Low-molecular-weight (LMW)-HA has been associated with the stimulation of keratinocyte proliferation, cellular migration, and angiogenesis, thereby supporting early regenerative and inflammatory responses. In contrast, high-molecular-weight (HMW)-HA contributes predominantly to skin hydration by forming a viscoelastic network that binds water molecules while also providing structural stability to the ECM and exerting anti-inflammatory properties^10-12^. Additionally, HA has been shown to attenuate oxidative stress and facilitate the repair of damaged tissues, reinforcing its significance in maintaining overall skin health.

This systematic review aims to examine the multifaceted effects of HA on the skin at the cellular level. By exploring the underlying molecular mechanisms and signaling pathways through which HA exerts its biological activity, we seek to provide a comprehensive and mechanistically informed overview of its role in skin physiology. Furthermore, we evaluate the current advancements in HA-based therapeutic strategies and discuss existing challenges regarding their clinical translation. Through this analysis, we aim to highlight the importance of HA in modern dermatological science and its potential to enhance skin rejuvenation, repair, and overall function.

## METHODS

A systematic literature review was conducted in accordance with the Preferred Reporting Items for Systematic reviews and Meta-Analyses (PRISMA) 2020 guidelines to ensure transparency and reproducibility. Three electronic databases—PubMed, Scopus, and Web of Science—were systematically searched to identify relevant studies investigating the cellular effects of HA on the skin.

### Search strategy

The search covered publications from January 2009 to February 2024. The following combination of Medical Subject Headings (MeSH) and free-text terms was used:

("hyaluronic acid" OR "HA") AND ("skin" OR "dermis" OR "epidermis") AND ("keratinocyte" OR "fibroblast" OR "hydration" OR "wound healing" OR "extracellular matrix").

Boolean operators (AND, OR) were applied to combine terms appropriately. All retrieved citations were exported into the EndNote X9 reference manager, and duplicates were removed.

### Inclusion criteria

- Peer-reviewed original research and review articles;
- Studies published in English;
- In vitro, in vivo, clinical, and review studies investigating the cellular or molecular mechanisms of HA on the skin;
- Articles addressing keratinocyte and fibroblast responses, ECM interactions, wound healing, oxidative stress, and inflammatory pathways.

### Exclusion criteria

- Editorials, conference abstracts, and case reports;
- Articles not focused on the cellular or molecular mechanisms of HA on the skin;
- Non-English language publications;
- Non-peer-reviewed content.

### Data extraction and synthesis

Relevant data were extracted using a standardized form, including information on study type, cell lines or tissue used, MW of HA, cellular outcomes (e.g., proliferation, migration, and collagen production), and signaling pathways involved. The results were synthesized qualitatively due to heterogeneity in experimental designs. Data extraction was performed independently by two authors. Any discrepancies were resolved by consensus.

### Preferred Reporting Items for Systematic reviews and Meta-Analyses flow diagram

**Figure 1:**
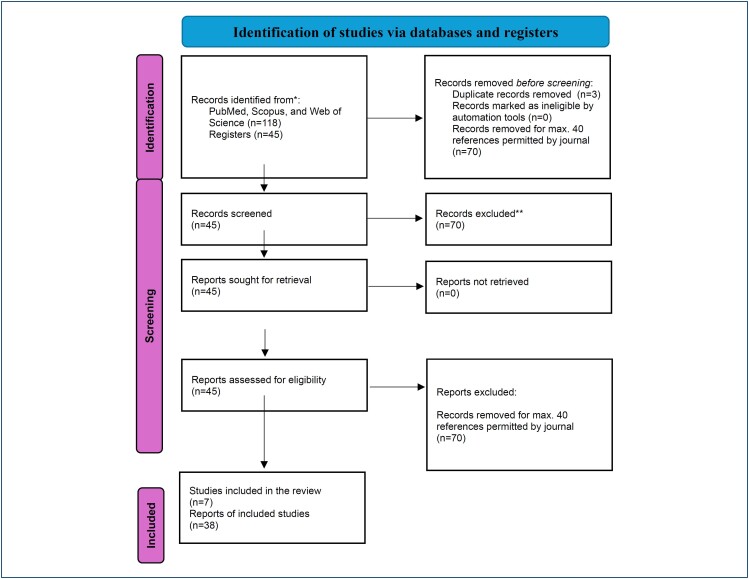
Preferred Reporting Items for Systematic reviews and Meta-Analyses 2020 flow diagram showing the study selection process for the systematic review of hyaluronic acid's effects on the skin at the cellular level. A total of 118 articles were identified across PubMed, Scopus, and Web of Science. After removing three duplicates, 115 studies were screened. All 115 studies were assessed for full-text eligibility, and 70 were excluded due to being published outside the 5-year window prioritized by the journal's reference policy. Consequently, 45 articles were included in the final qualitative analysis. *Databases searched included PubMed, Scopus, and Web of Science (n=118) and clinical trial or systematic review registers (n=45). **Exclusion reasons included studies with irrelevant outcomes, non-cutaneous models, reviews without cellular-level data, or insufficient methodological detail.

The study selection process is visually summarized in Figure 1, a Preferred Reporting Items for Systematic reviews and Meta-Analyses (PRISMA) flow diagram that outlines the number of records identified, screened, assessed for eligibility, and included in the review, along with reasons for exclusions. Due to the heterogeneity of included studies in terms of design, outcome measures, and MW of HA formulations, a meta-analysis was not performed.

## RESULTS

### Risk-of-bias assessment

To assess the methodological quality and potential bias of the included studies, we applied the Risk of Bias in Systematic Reviews (ROBIS) tool, which is designed to evaluate the risk of bias in systematic reviews through a structured three-phase approach: (1) assessment of relevance, (2) identification of concerns regarding study identification and selection, and (3) judgment of overall risk of bias. Additionally, for experimental and clinical studies included in our analysis, the Grading of Recommendations Assessment, Development, and Evaluation (GRADE) approach was used to evaluate the certainty of the evidence based on five domains: risk of bias, inconsistency, indirectness, imprecision, and publication bias. The overall confidence in the evidence was categorized as high, moderate, low, or very low. The risk-of-bias assessment was conducted jointly by the two authors of this review. Any uncertainties or disagreements were resolved through consensus (Table 1).

**Table 1:** Risk of bias and certainty of evidence summary for included studies.

Study (author)	Type of study	Main focus	ROBIS assessment	GRADE certainty	Notes
Hynnekleiv et al.^1^	Clinical	HA in dry eye disease	Low	High	Peer-reviewed RCT with well-described methods and clear endpoints
Huang et al.^3^	Clinical	HA in photodynamic facial therapy	Moderate	Moderate	Pilot-level data; small sample size but well controlled
Muhammad et al.^8^	Clinical (RCT)	HA of different MWs in xerosis cutis	Low	High	Double-blinded RCT with statistical power and clear results
Drozdova et al.^10^	In vitro	HA-based hydrogels in tissue engineering	Moderate	Moderate	Limited to in vitro conditions; reproducible methods
Karam et al.^12^	In vitro	Effect of HA's MW on angiogenesis	Low	Moderate	Clear experimental design; lacks in vivo confirmation
Wang et al.^18^	In vivo	Triple-responsive HA hydrogel for diabetic wounds	Low	High	Strong in vivo data; comprehensive biological endpoints
Chang et al.^19^	In vivo	Bioengineered HA in tissue regeneration	Low	Moderate	Animal model validated; some variability in methodology
Wang et al.^33^	In vivo	HA–collagen hydrogel for vascularization	Low	High	Methodologically sound, appropriate controls and analysis
Hu et al.^28^	In vitro	Effects of HA's MW on keratinocyte inflammation	Moderate	Moderate	Mechanistic insights; lacks translational modeling

ROBIS: Risk of Bias in Systematic Reviews; GRADE: Grading of Recommendations Assessment, Development, and Evaluation; HA: hyaluronic acid; MW: molecular weight; RCT: randomized controlled trial.

#### Interaction with keratinocytes

HA plays a pivotal role in epidermal homeostasis by promoting keratinocyte proliferation and differentiation. Through CD44 receptor-mediated signaling, HA enhances cell migration and supports barrier function^13-15^.

#### Effects on fibroblasts

HA stimulates fibroblast activity, increasing the production of collagen, elastin, and ECM proteins. This contributes to skin firmness, elasticity, and resistance to mechanical stress^16,17^.

#### Wound healing mechanisms

HA is involved in every stage of wound healing, from inflammation to tissue remodeling. It regulates immune responses, facilitates cell migration, and supports angiogenesis^18,19^.

#### Anti-inflammatory and antioxidant properties

HA modulates inflammatory pathways by reducing pro-inflammatory cytokines such as tumor necrosis factor-α (TNF-α) and interleukin-6 (IL-6) while also neutralizing reactive oxygen species (ROS), thereby protecting against oxidative stress^20-22^.

#### Clinical applications

HA is widely used in dermatology for dermal fillers, topical serums, and skin-rejuvenating treatments. It is effective in treating conditions such as xerosis, atopic dermatitis, and post-procedural skin recovery^23,24^.

#### Cellular signaling pathways involving hyaluronic acid

HA exerts its effects at the cellular level primarily through interactions with cell surface receptors such as CD44, receptor for hyaluronan-mediated motility (RHAMM), and Toll-like receptors (TLR2/4)^25^.

#### CD44 signaling

CD44 is a principal HA receptor expressed on keratinocytes and fibroblasts. Upon HA binding, CD44 activates downstream signaling cascades, including the MAPK/ERK and PI3K/Akt pathways. These pathways regulate cell proliferation, migration, and survival, contributing to wound healing and skin regeneration^25,26^.

#### Receptor for hyaluronan-mediated motility signaling

RHAMM, another HA-binding receptor, mediates cell motility and tissue remodeling through activation of intracellular signaling cascades like JAK/STAT and focal adhesion kinase, which are involved in fibroblast migration and collagen synthesis^27^.

#### Toll-like receptors

HA fragments can interact with TLR2/4, initiating immune responses in the skin. While HMW-HA has anti-inflammatory properties, LMW-HA can activate inflammatory responses by engaging these receptors, which is relevant in wound healing and pathological conditions such as psoriasis^28^.

#### Hyaluronic acid degradation mechanisms

HA undergoes continuous turnover in the skin, primarily regulated by enzymatic degradation and oxidative processes.

#### Hyaluronidases

The primary enzymes responsible for HA breakdown are HYALs, particularly HYAL1 and HYAL2. HYAL1 degrades HA into small oligosaccharides, whereas HYAL2 initiates its fragmentation into intermediate-sized fragments. This degradation affects skin hydration and elasticity, impacting aging and wound healing processes^29^.

#### Oxidative stress-induced degradation

ROS generated by ultraviolet (UV) radiation, pollution, and inflammatory processes contribute to HA breakdown. The oxidative degradation of HA leads to loss of skin moisture and increased susceptibility to wrinkling and sagging^30^.

#### pH-dependent degradation

HA stability is influenced by skin pH. Under acidic or highly alkaline conditions, HA undergoes hydrolysis, affecting its functional properties in skin care formulations and physiological conditions^31^.

**Figure 2:**
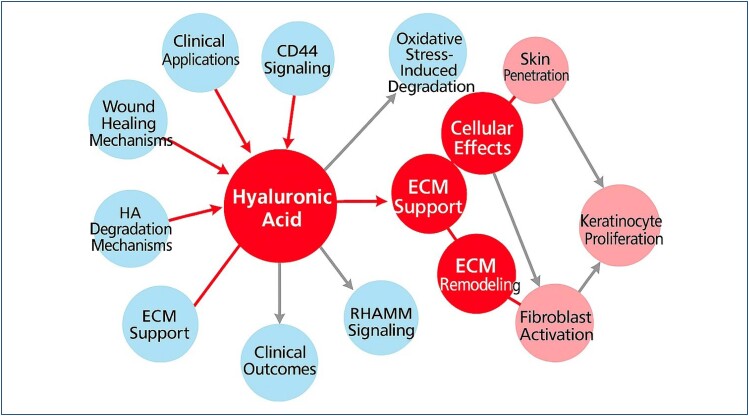
Schematic representation of the cellular-level mechanisms and effects of hyaluronic acid on skin physiology. Hyaluronic acid interacts with key biological pathways, including CD44 and receptor for hyaluronan-mediated motility signaling, extracellular matrix support and remodeling, and oxidative stress-induced degradation. These interactions collectively contribute to processes such as wound healing, fibroblast activation, keratinocyte proliferation, and skin penetration. Red circles in the diagram represent direct mechanistic roles of hyaluronic acid, light blue circles indicate upstream physiological pathways influenced by hyaluronic acid, and pink circles reflect downstream cellular responses. This integrated visual highlights hyaluronic acid's multifaceted role in maintaining skin homeostasis, promoting tissue repair, and supporting clinical applications in dermatology.

To summarize the multifaceted biological actions of HA in skin physiology, an integrated diagram was constructed. The schematic representation illustrates how HA interacts with key molecular components such as CD44, RHAMM, and TLR2/4 while also highlighting its susceptibility to oxidative and enzymatic degradation. These mechanistic pathways converge toward functional effects, including keratinocyte proliferation, fibroblast activation, ECM support, and ultimately improved clinical outcomes. This dual-layered visualization underscores HA's central role in both upstream cellular signaling and downstream regenerative processes involved in skin health (Figure 2).

## DISCUSSION

At the cellular level, HA supports the optimal function of keratinocytes by forming a protective barrier that reduces transepidermal water loss. By enhancing ECM hydration, it contributes to skin elasticity, smoothness, and structural resilience. These properties are especially significant as HA levels decline with age, leading to dryness, fine lines, and impaired regeneration^32^. When applied topically, HA interacts with the stratum corneum to draw moisture into the skin, while its presence in deeper layers helps regulate water–solute balance within the dermis, supporting overall skin homeostasis.

HA is also a central mediator in wound healing, contributing to inflammation regulation, cell migration, angiogenesis, and ECM remodeling^33^. Following injury, increased HA levels at the wound site facilitate immune cell recruitment and promote fibroblast and endothelial cell activity, accelerating tissue repair. Through its interaction with CD44 and RHAMM receptors, HA enhances cellular proliferation and matrix production^34^. The biological activity of HA during healing is closely linked to its MW: LMW-HA is predominant in the early inflammatory phase and promotes immune activation and cell migration, whereas HMW-HA supports resolution and structural stabilization^35^.

Inflammation is a critical factor in both the aging process and the response to skin injury. HA, through its interaction with various receptors, plays a vital role in modulating inflammation at the cellular level. During the inflammatory phase of wound healing, LMW-HA acts as a pro-inflammatory mediator, promoting the activation of immune cells such as macrophages and neutrophils. However, once the inflammatory process begins to resolve, HMW-HA helps switch off the inflammatory response by binding to CD44 and inhibiting the release of pro-inflammatory cytokines. This anti-inflammatory effect of HMW-HA is essential not only for wound healing but also for maintaining healthy skin by preventing chronic low-grade inflammation, which is a hallmark of many skin conditions, including acne, rosacea, and psoriasis^36^.

Additionally, HA's ability to modulate inflammation extends to its influence on oxidative stress. Oxidative damage to skin cells accelerates aging and impairs skin barrier function. By reducing oxidative stress and promoting cellular antioxidant defense mechanisms, HA contributes to the protection of skin cells from damage caused by free radicals, UV radiation, and environmental pollutants.

Fibroblasts are the primary cell type responsible for producing collagen, elastin, and other ECM components in the dermis. The interaction between HA and fibroblasts is essential for maintaining the structural integrity of the skin. HA regulates fibroblast behavior by enhancing their migration, proliferation, and ECM production. Furthermore, HA influences the synthesis and degradation of collagen, which is fundamental to maintaining skin elasticity and firmness.

HA directly affects fibroblast activity by interacting with CD44 and RHAMM, which are involved in cell adhesion, migration, and proliferation. In the context of wound healing, HA helps recruit fibroblasts to the wound site, where they contribute to collagen production and the deposition of other ECM components that are necessary for tissue repair. This process also involves the regulation of matrix metalloproteinases (MMPs), enzymes that degrade the ECM during remodeling. HA helps balance ECM turnover by modulating MMP activity, ensuring that the repair process is efficient and not excessive^37^.

Skin aging is a complex process influenced by both intrinsic (genetic) and extrinsic (environmental) factors. The decline in HA levels with age is one of the key factors contributing to the visible signs of aging, such as wrinkles, loss of volume, and reduced skin elasticity. As HA content decreases, the skin becomes drier and more prone to damage, with a reduced capacity for regeneration. The degradation of HA in the dermis leads to a thinner skin matrix and a weakened ability to retain moisture, which accelerates the appearance of fine lines and wrinkles^38^.

Interestingly, topical and injectable forms of HA have been shown to have rejuvenating effects by replenishing the skin's HA stores and improving its overall hydration. Injectable HA dermal fillers are commonly used in esthetic dermatology to restore volume, smooth wrinkles, and enhance facial contours. These treatments provide both immediate cosmetic benefits and long-term improvements by stimulating the skin's fibroblasts to produce collagen and elastin.

A critical distinction must be made between the biological effects of HA based on its MW. HMW-HA and LMW-HA exhibit markedly different physiological properties, particularly in relation to inflammation and tissue remodeling. While HMW-HA plays an anti-inflammatory role by stabilizing the ECM and inhibiting inflammatory cytokine release via CD44-mediated pathways, LMW-HA is frequently associated with pro-inflammatory effects, especially during the early stages of tissue injury. Specifically, LMW-HA can engage TLR2/4 and activate downstream signaling cascades that promote the release of TNF-α and IL-6, which are critical mediators of acute inflammation^28^. This dualistic behavior underscores the need for MW-specific formulations in therapeutic applications. For example, in wound healing, the transient presence of LMW-HA may stimulate early immune responses and cell migration, whereas the subsequent predominance of HMW-HA contributes to inflammation resolution and matrix stabilization^35^. Thus, understanding and controlling the MW profile of HA formulations is essential to optimize clinical efficacy and reduce unintended inflammatory responses.

Despite the widespread clinical use of HA in topical formulations, dermal fillers, and regenerative medicine, several limitations must be acknowledged in translating its biological effects into sustained therapeutic outcomes. One significant challenge is the limited bioavailability of topically applied HA, as its large molecular size often restricts penetration beyond the stratum corneum. Although advances such as microneedling, liposomal encapsulation, and LMW formulations have improved transdermal delivery^32^, maintaining effective concentrations in deeper skin layers remains difficult. In the context of injectable fillers, while HMW-HA-based gels offer immediate volumizing effects and stimulate fibroblast activity^37^, their longevity is influenced by enzymatic degradation (e.g., by HYALs) and oxidative stress^29,30^. Adverse effects, including granuloma formation, delayed hypersensitivity reactions, and vascular complications, although rare, have been reported in post-marketing surveillance and clinical studies^24^. These concerns highlight the need for standardized formulations, long-term safety data, and individualized treatment planning. Moreover, variability in patient response based on age, skin condition, and immune status further complicates outcome predictability. Therefore, while HA holds substantial clinical promise, its application requires a critical balance between efficacy, stability, and patient safety.

Despite its promising effects on skin health, the use of HA in dermatology faces several challenges. The MW of HA plays a significant role in determining its biological effects, and the ability to control its molecular structure for specific therapeutic outcomes remains a challenge. Additionally, the long-term effects of HA application, particularly in injectable forms, are not yet fully understood, and further research is needed to assess the potential risks and benefits of prolonged HA treatments^39^.

Moreover, the development of HA-based products for topical application often faces limitations regarding the permeability of the skin barrier. Ongoing studies are exploring ways to enhance the penetration of HA into deeper skin layers and improve its bioavailability^40^.

Hyaluronan is increasingly recognized as a dynamic component of the ECM with a surprisingly rapid turnover rate, despite its structural role in connective tissues. According to Laurent and Fraser, hyaluronan is synthesized at the plasma membrane and secreted into the pericellular space, where it participates in cellular regulation, water homeostasis, and mitotic activity through receptor-mediated interactions, particularly involving CD44. Its degradation is primarily mediated by HYALs or via systemic clearance through the lymphatic system and hepatic endothelial cells. These mechanisms not only regulate HA levels in tissue microenvironments but also link its accumulation or depletion to pathological conditions such as inflammation, fibrosis, and interstitial edema. The authors further emphasized hyaluronan's relevance in clinical contexts, noting its application in ophthalmic surgery and its potential as a biomarker in hepatic and rheumatologic diseases^41^.

In recent years, the clinical application of HA dermal fillers has expanded significantly, prompting the development of expert consensus recommendations to ensure safe and effective use. Proper product selection based on rheological properties and the anatomical region of treatment is fundamental to achieving natural esthetic outcomes without compromising safety. These recommendations emphasize the importance of appropriate injection techniques, depth control, and anatomical precision to reduce complications such as vascular occlusion or delayed inflammatory responses. Furthermore, the integration of HA-based strategies with an understanding of its ECM interactions and hydration-promoting effects enhances both the functional and cosmetic benefits observed in clinical settings. Together, these clinical insights highlight the translational value of HA from molecular research to evidence-based dermatological practice^42^. However, it should also be noted that excessive or repeated stimulation of fibroblasts by HA, particularly in chronic inflammatory environments or with high-dose applications, may paradoxically contribute to fibrotic responses and excessive ECM deposition. Such findings underscore the importance of MW-specific formulations and context-driven clinical decision-making to avoid unintended tissue remodeling effects^43^.

A recent experimental study investigating the expression of HA in the mouse uterine horns across the estrous cycle revealed that HA levels fluctuate in accordance with hormonal phases. Using both fluorometric quantification and immunohistochemistry, the researchers demonstrated that HA content and CD44 expression peaked during diestrus, with strong localization beneath the luminal epithelium and in connective tissues adjacent to the myometrium. These findings suggest that estrogen and progesterone modulate HA distribution in reproductive tissues and highlight the potential role of HA in endometrial remodeling and embryo implantation^44^.

Similar to other endogenous biomolecules with antioxidant capacity, such as melatonin, HA plays a significant role in maintaining skin redox homeostasis. A recent systematic review examining melatonin's effects in rat models revealed consistent upregulation of key antioxidant enzymes, including superoxide dismutase, glutathione peroxidase, and catalase, alongside a reduction in oxidative stress markers such as malondialdehyde^45^. These findings reinforce the concept that antioxidant modulation is a central mechanism in tissue protection. In parallel, HA has been shown to reduce ROS, stabilize the ECM, and enhance cellular antioxidant responses, particularly under conditions of environmental stress and photoaging. The convergence of evidence from both melatonin- and HA-based models supports the rationale for targeting oxidative stress pathways in the development of regenerative and protective skin therapies.

## CONCLUSION

HA is an essential biomolecule in skin biology, influencing a wide range of cellular processes that are crucial for maintaining skin health and function. Its effects on hydration, wound healing, inflammation modulation, and ECM remodeling highlight its multifaceted role in dermatology. Moreover, HA's ability to support skin regeneration and rejuvenation makes it a valuable therapeutic tool in both clinical and cosmetic applications. As research continues to uncover the molecular mechanisms underlying HA's effects, its potential to improve skin health and treat a variety of skin conditions will likely expand, offering new avenues for non-invasive treatments and advanced skincare solutions. With continued advancements in molecular formulations, delivery systems, and clinical validation, HA is poised to become a cornerstone of personalized dermatological interventions in both therapeutic and esthetic medicine.

## Data Availability

The datasets generated and/or analyzed during the current study are available from the corresponding author upon reasonable request.
